# Bilateral Median Nerve and Brachial Artery Variations in the Arm: A Case Report

**DOI:** 10.7759/cureus.51350

**Published:** 2023-12-30

**Authors:** Mathangi Rajaram-Gilkes, Julian Burwell, Kelly Barr, Daniel Marcincavage, Kristi Fung, Ene Chukwuemeka

**Affiliations:** 1 Medical Education, Geisinger Commonwealth School of Medicine, Scranton, USA

**Keywords:** bilateral median nerve anomaly, radial artery in cubital fossa, median nerve variations, high division of brachial artery, anatomical variation

## Abstract

During clinical practice, it is essential for physicians to have a sound knowledge of vascular and nerve variations. Patients who present with various clinical signs and symptoms need to be thoroughly investigated with anatomic variations in mind to prevent misdiagnosis. Most nerve variations are related to their formation or their course and are frequently associated with variability of structures that surround them. These structures most commonly include blood vessels, ligaments, and muscles. Such variations should be foremost in a physician’s mind when analyzing clinical symptoms. This will aid in accurate diagnosis, and if surgical intervention is warranted, such awareness would minimize intraoperative errors. In this case study, the striking absence of median nerve and brachial artery within the cubital fossa bilaterally led to the discovery of pronator teres originating from the distal third of the humerus, associated with the bifurcation of the brachial artery at the middle third of the humerus into the ulnar and radial arteries. The median nerve ran beneath the pronator teres along with the ulnar artery and was thereby absent at the cubital fossa. Such variations observed bilaterally have not yet been reported in the literature. Knowledge of such variations can be very profound as this region involves surgical significance for several conditions, such as creation of arteriovenous fistulas (AVFs) for hemodialysis, treatment of supracondylar and radial head fractures, and cubital tunnel syndrome.

## Introduction

The intricate network of nerves and arteries within the human body plays a crucial role in maintaining optimal motor and sensory function. Understanding the variations in the trajectory and relationships of the median nerve and brachial artery is important for clinicians, surgeons, and anatomists alike, as these anomalies can impact diagnostic accuracy, surgical procedures, and overall patient care. Our investigation focuses on the unique anatomic variations observed in two upper limbs with median nerve and brachial artery variations.

The median nerve is a terminal branch of the brachial plexus, originating from the lateral and medial cords. It descends along the medial aspect of the arm, lateral to the brachial artery in the upper part, and crosses over to the medial side and contacts the brachialis muscle. It then descends into the cubital fossa, where it lies deep to the bicipital aponeurosis. The median nerve has no branches in the axilla or arm but provides articular branches to the elbow joint. The brachial artery is the main arterial source of the upper limb. The subclavian crosses the outer border of the first rib to continue as the axillary artery, which changes name to brachial at the lower border of the teres major [1]. The cubital fossa, located at the elbow, has a triangular shape and is bounded by an imaginary line joining the medial and lateral epicondyles at the base; the medial border is formed by the pronator teres and the lateral border by the brachioradialis muscles. The proximal aspect of the floor of the cubital fossa is formed by the brachialis, while the supinator contributes to the distal half. The roof is essentially composed of the bicipital aponeurosis, superficial to which are the subcutaneous veins covered by skin. At the apex of the cubital fossa, the median nerve passes deep into the forearm muscles between the humeral and ulnar heads of the pronator teres. The brachial artery divides as the radial and ulnar arteries at this point as well [2].

The median, radial, and ulnar nerves mainly innervate the forearm muscles, and the radial and ulnar arteries provide vascular supply. The median nerve innervates most of the forearm flexors as it passes through the forearm, and as it emerges from the carpal tunnel, it innervates a few intrinsic muscles of the hand. The deep flexor compartment muscles are innervated by the anterior interosseous nerve, which is a branch of the median nerve. The radial and ulnar arteries arising from the bifurcation of brachial artery at the apex of cubital fossa provide the major blood supply of the forearm and hand [3].

The observations in our case indicate bilateral variations in the origin of the pronator teres, leading to the altered course of the median nerve and bifurcation of the brachial artery into radial and ulnar branches at the middle third of the humerus. These deviations can be traced back to the development of the limb buds in the fourth week of embryological development [4], where the interplay of genetic and environmental factors contribute to their final anatomical arrangement [5,6]. Understanding these developmental processes is pivotal for appreciating the variations observed in the adult anatomy of the median nerve and brachial artery. 

Studies done on median nerve formation indicate variations in the formation of the median nerve at the brachial plexus level. They indicate variations in communications between the medial and lateral cords and more than two contributions to median nerve formation [7,8]. Some studies indicate an incidence of high divisions in the brachial artery ranging between 5% and 14% of the cadavers [9,10]. Rarely were these variations demonstrated bilaterally and never within the same specimen. Our exploration into this unique case hopes to shed light on the preponderance of anatomical variations and their clinical relevance, offering healthcare professionals and anatomists a different perspective on bilateral variations.

## Case presentation

In the Geisinger Commonwealth School of Medicine gross anatomy lab, upper limb dissections were performed on 20 cadavers during year one of the medical curriculum. During dissection, bilateral variations of the brachial artery and median nerve were observed in an elderly female cadaver. Having described the normal formation, course, and basic distribution of the nerves and arteries of the upper limb in the introduction section, we bring forward the observations in our case here. The images shown are arranged in a sequential manner to show variations on both sides regarding the observations in the arm, elbow, and forearm regions.

Observations in the arm

The brachial plexus formation seemed to be normal on both sides. All branches from the cords and terminal branches were identifiable. The axillary artery ran between the medial and lateral cords and was superior to the posterior cord. All branches from the axillary arteries seemed normal. Past the teres major, the brachial artery was observed to give rise to the profunda brachii artery. Just past this point, at the level of the middle third of the humerus, the brachial artery was observed to bifurcate into two arteries of robust diameter. The medially located ulnar artery seemed to run along with the median nerve as it crossed from the lateral to the medial aspect and the lateral branch descended to the cubital fossa to run beneath the bicipital aponeurosis. The median nerve was observed to pass under the pronator teres muscle, which originated at the junction of the middle and distal third of the humerus. Normally, this muscle originates from a common flexor tendon from the medial epicondyle of the humerus. All the above-mentioned variations were observed bilaterally.

**Figure 1:**
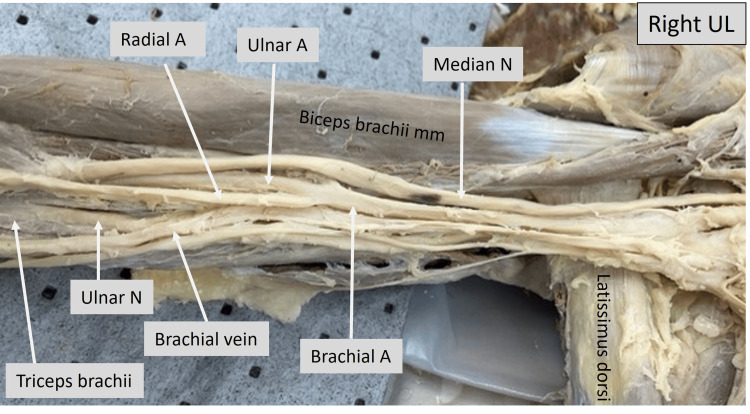
Cadaveric image of the right arm. Structures of interest: a) Level of brachial A bifurcation, b) ulnar A, c) ulnar N, d) median N, e) pronator teres mm, f) biceps brachii mm. A - artery, N -nerve, mm - muscle, UL - upper limb.

In the image of the right upper limb shown below (Fig. 1), the upper part of the biceps brachii can be seen. The median nerve and brachial artery can be observed running along the medial aspect of the biceps up to the level where the profunda brachii artery is given off. The proximity to the insertion of the latissimus dorsi and pectoralis major on the humerus gives an approximate landmark as to the distance at which the brachial artery bifurcates into the radial and ulnar arteries. The ulnar nerve was observed to enter the medial head of triceps.

**Figure 2:**
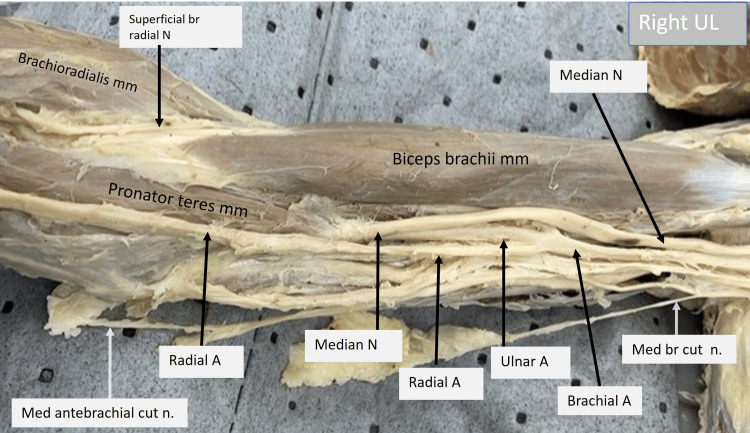
Cadaveric image of the right arm and elbow. Structures of interest: a) level of brachial A bifurcation, b) ulnar A, c) ulnar N, d) median N, e) pronator teres mm, f) biceps brachii mm. A - artery, N - nerve, mm - muscle, UL - upper limb.

The image of the right upper limb shown below (Fig. 2) focuses on the arm and elbow regions. The course of the median nerve, brachial artery, and its branches can be identified here. The following can be identified as variations: the pronator teres muscle takes a high origin from the lower third of the humerus on the anterior aspect. The median nerve can be observed to run beneath this humeral head of the pronator teres muscle, thereby being absent in the cubital fossa. The brachial artery is observed to give rise to its terminal branches, such as the radial and ulnar arteries at a mid humerus level. The ulnar artery seems to run along with the median nerve beneath the pronator teres muscle. The medial brachial and antebrachial cutaneous nerves have been labeled and are within the normal anatomical location and distribution. 

**Figure 3:**
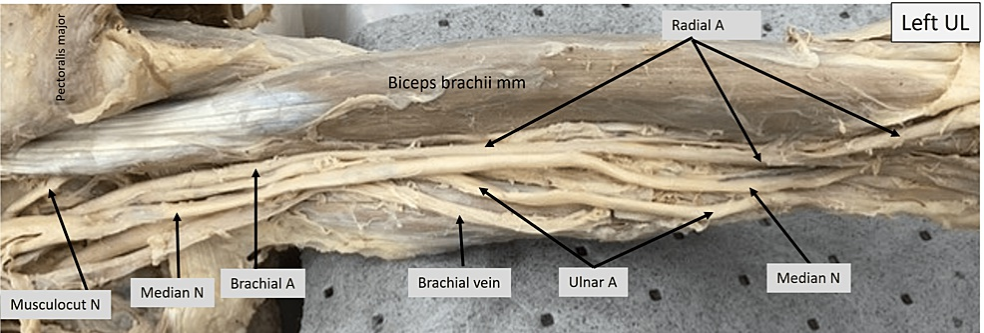
Cadaveric image of the left arm. Structures of interest: a) level of brachial A bifurcation, b) ulnar A, c) median N, d) pronator teres mm, e) biceps brachii mm. A - artery, N - nerve, mm - muscle, UL - upper limb.

In the image of the left upper limb shown below (Fig. 3), the arm muscles and neurovascular structures can be visualized. The tendinous origin of the biceps can be seen at the left and its lower end toward the right. Medial to it, the median nerve can be seen running along with the brachial artery and ulnar nerve. The brachial artery divides into the radial and ulnar arteries approximately about the middle third of the humerus. The median nerve was observed to run under the pronator teres, which originates from the distal third of the humerus, on its medial aspect in our case. The ulnar artery was observed to run along with the median nerve. The radial artery continues to run beneath the bicipital aponeurosis on to the cubital fossa. 

**Figure 4:**
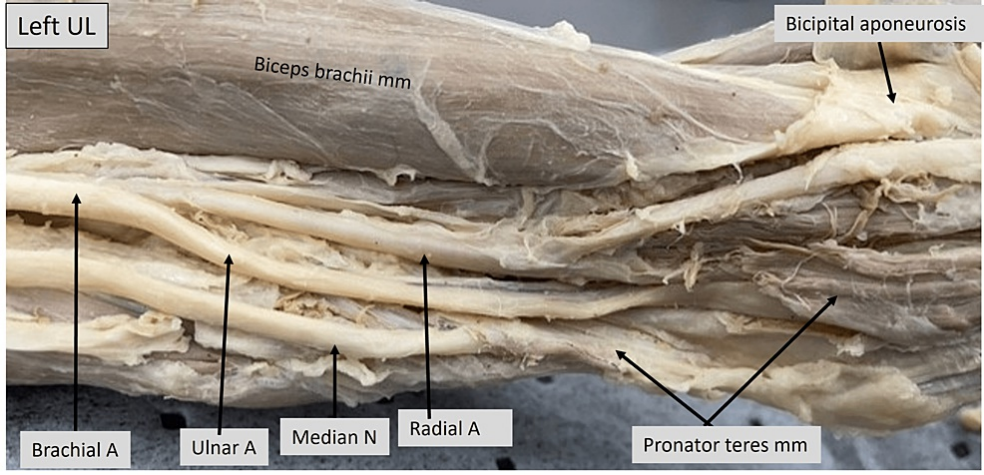
Cadaveric image of the left arm and elbow. Structures of interest: a) level of brachial A bifurcation, b) ulnar A, c) ulnar N, d) median N, e) pronator teres mm, f) biceps brachii mm, g) bicipital aponeurosis. A - artery, N - nerve, mm - muscle, UL - upper limb.

The image shown below (Fig. 4) is a closer view of the left upper limb showing the bifurcation of the brachial artery. The radial artery can be seen entering the cubital fossa beneath the reflected bicipital aponeurosis. The median nerve can be observed to run along with the ulnar artery and disappear under the humeral head of the pronator teres muscle. 

Observations at the cubital fossa

At the cubital fossa, beneath the bicipital aponeurosis, instead of the median nerve and brachial artery, the only content to course through was the radial artery. This was the case bilaterally. The pronator teres muscle was dissected. The humeral head was observed to originate from the distal third of the shaft of the humerus and from the medial epicondyle. The ulnar head originated from the coronoid process. Distally, the muscle was attached to the lateral surface of the mid radius.

**Figure 5:**
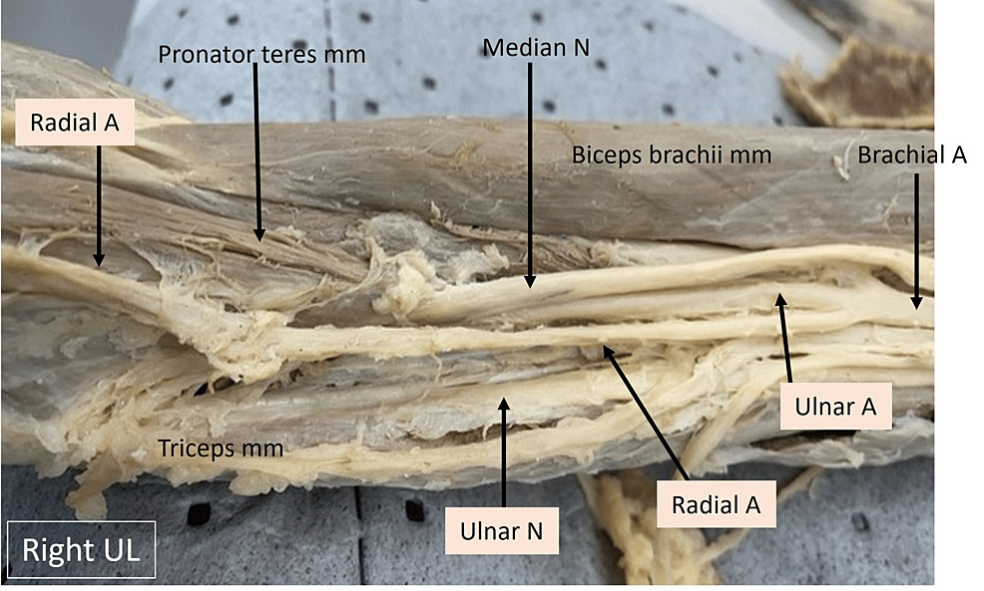
Cadaveric image of the right upper limb focusing on the elbow region. Structures of interest: a) level of brachial A bifurcation, b) ulnar A, c) ulnar N, d) median N, e) pronator teres mm, f) biceps brachii mm. A - artery, N - nerve, mm - muscle.

The image below (Fig. 5) is from the right upper limb showing a close view of the bifurcation of the brachial artery. The radial artery can be seen running into the cubital fossa, while the ulnar artery accompanies the median nerve.

**Figure 6:**
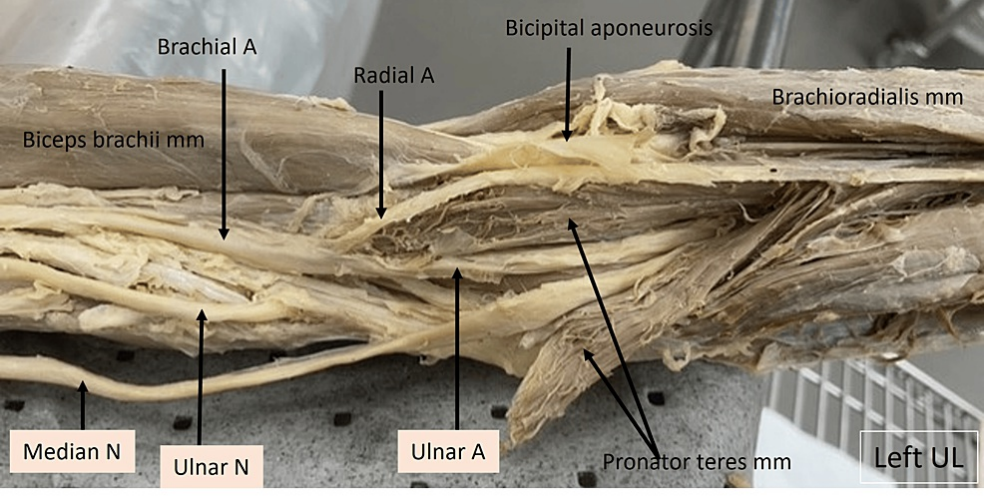
Cadaveric image of the left upper limb focusing on the elbow region. Structures of interest: a) level of brachial A bifurcation, b) ulnar A, c) ulnar N, d) median N, e) pronator teres mm, f) biceps brachii mm, g) bicipital aponeurosis, h) brachioradialis mm. A - artery, N - nerve, mm - muscle.

In the image below (Fig. 6), the elbow and proximal flexor aspect of the forearm of the left upper limb is shown. The radial artery can be observed to run beneath the bicipital aponeurosis. The pronator teres muscle has been dissected here. A part of the humeral head can be seen reflected off from the distal third of the shaft of the humerus to show the passage of the median nerve and ulnar artery. These two structures can be seen descending into the cubital area to pass between the humeral and ulnar heads of the pronator teres muscle. The humeral head of the pronator teres can be observed to originate just beneath the bifurcation of the brachial artery.

Observations in the forearm

The radial artery was observed to run through the cubital fossa, emerged below the bicipital aponeurosis, and continued its journey to the wrist along the medial border of the brachioradialis muscle. When the humeral and ulnar heads of the pronator teres were dissected, the median nerve and ulnar artery were observed to enter the deeper aspect of the flexor compartment. At the proximal end of the interosseus membrane, the common interosseus artery emerged from the ulnar artery and divided as the anterior and posterior interosseus arteries. After this branch, the ulnar artery continued to run toward the ulnar nerve, and they travelled together beneath the flexor carpi ulnaris (FCU) and emerged at the wrist to run a normal course. The pattern was observed to be the same bilaterally. The distribution of blood vessels and other nerves were observed to be normal in the hands.

**Figure 7:**
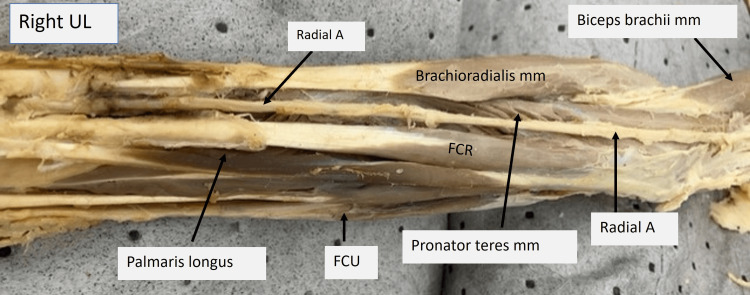
Cadaveric image of the right upper limb focusing on the elbow and forearm. Structures of interest: a) radial A, b) brachioradialis mm, c) flexor carpi ulnaris (FCU), d) palmaris longus, e) pronator teres mm, f) biceps brachii mm, g) bicipital aponeurosis, h) flexor carpi radialis (FCR). A - artery, N - nerve, mm - muscle, UL - upper limb.

The image of the right upper limb below (Fig. 7), the distal end of the arm, the elbow, and the flexor aspect of the forearm can be seen. Once the radial artery originated from the brachial artery at the mid-arm level, it ran within the cubital fossa on the surface of the brachialis and supinator. At the apex, it continued to run the normal course within the forearm and hand. This artery was the only content of the cubital fossa. The median nerve and brachial artery, which are normally found within the cubital fossa, were absent. 

**Figure 8:**
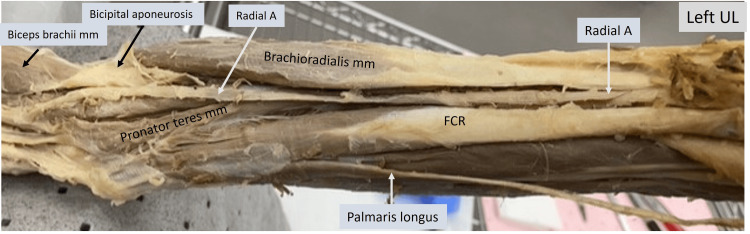
Cadaveric image of the left upper limb focusing on the elbow and forearm. Structures of interest: a) radial A, b) brachioradialis mm, c) flexor carpi radialis (FCR), d) palmaris longus, e) pronator teres mm, f) biceps brachii mm, g) bicipital aponeurosis. A - artery, N - nerve, mm - muscle, UL - upper limb.

The image of the left limb (Fig. 8) shows the elbow and flexor aspect of the forearm. Under the reflected bicipital aponeurosis, the radial artery can be seen running along the medial border of the brachioradialis muscle up to the radial aspect of the wrist. The median nerve is absent within the cubital fossa. 

**Figure 9:**
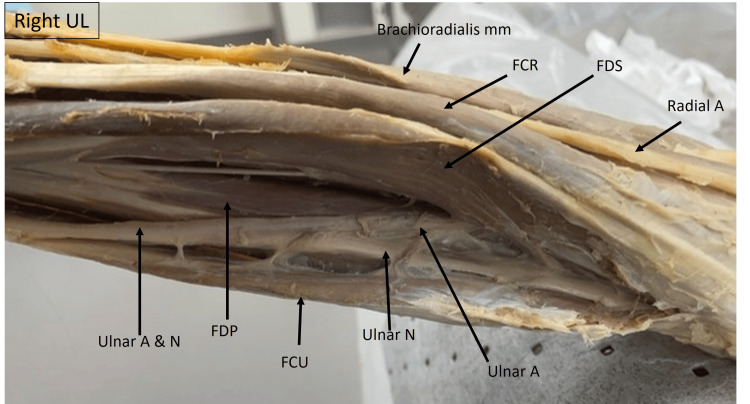
Cadaveric image of the right upper limb focusing the medial aspect of the forearm. Structures of interest: a) ulnar A, b) ulnar N, c) brachioradialis mm, d) radial A, e) flexor carpi ulnaris (FCU), d) flexor digitorum superficialis (FDS), e) flexor digitorum profundus (FDP), f) flexor carpi ulnaris (FCU). A - artery, N - nerve, mm - muscle, UL - upper limb.

The image below (Fig. 9) is that of the right upper limb, showing the forearm from a medial view. The ulnar artery can be seen to emerge beneath the FDS and runs along with the ulnar nerve as a neurovascular bundle beneath the FCU.

**Figure 10:**
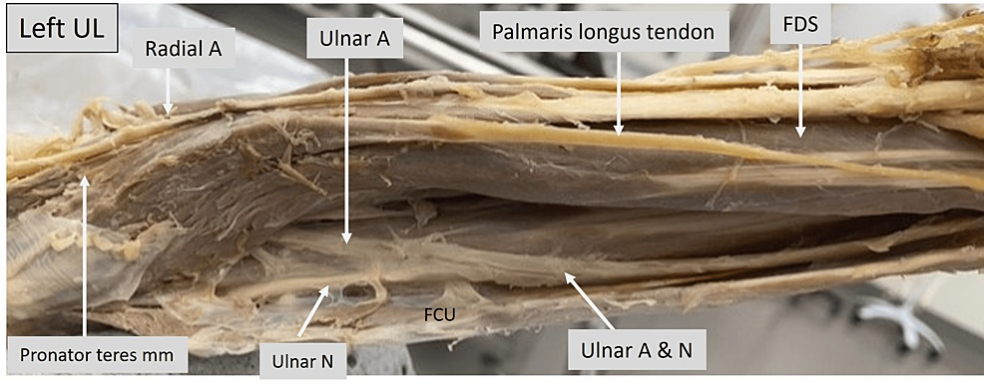
Cadaveric image of the left upper limb focusing on the medial aspect of the forearm. Structures of interest: a) radial A, b) flexor carpi ulnaris (FCU), c) palmaris longus tendon, d) pronator teres mm, e) flexor digitorum superficialis (FDS), f) ulnar A, g) ulnar N. A - artery, N - nerve, mm - muscle, UL - upper limb.

The image below (Fig. 10) is that of the left upper limb, showing the forearm from a medial view. The ulnar artery can be seen to emerge beneath the FDS and runs along with the ulnar nerve as a neurovascular bundle beneath the FCU. The pronator teres and FDS muscles are retracted for a clear view.

**Figure 11:**
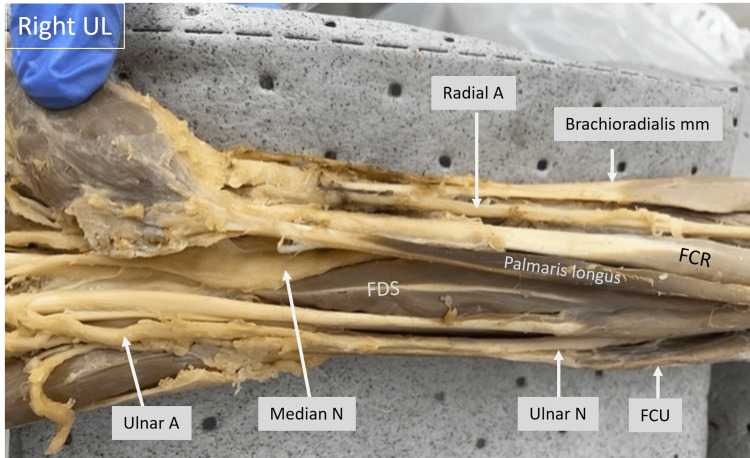
Cadaveric image of the right upper limb focusing on the distal forearm and wrist regions. Structures of interest: a) radial A, b) ulnar A, c) median N, d) brachioradialis mm, e) flexor carpi ulnaris (FCU), f) flexor carpi radialis (FCR), g) palmaris longus tendon. A - artery, N - nerve, mm - muscle, UL - upper limb.

In the image of the right upper limb shown below (Fig. 11), the distal forearm muscles, nerves, and vessels can be identified. The median nerve can be identified to emerge lateral to the FDS and can be seen entering the carpal tunnel. The ulnar artery and ulnar nerve can be seen emerging medial to the FCU and entering the wrist area. The branches and course of these were noted to be normal. The radial artery can also be identified here. 

## Discussion

The bilateral observation of the proximal origin of the pronator teres from the shaft of the humerus and the absence of the median nerve and brachial artery within the cubital fossa make us contemplate on the clinical implications of such variations. Although the radial and ulnar arteries and the median nerve were observed to have less significant variations in the forearm, there are several clinical conditions to consider. The elbow can be a critical place for clinical procedures, such as venipuncture, creation of arteriovenous fistulas (AVFs), surgical procedures such as cubital tunnel decompression, medial epicondylectomy, reduction and percutaneous pinning of supracondylar fractures, and repair of ruptured ligaments, to name a few. 

Different types of formation of the median nerve have been at the forefront of reported instances of median nerve anomalies within the upper arm. Uzun and Seelig [11] reported that the median nerve was formed by four roots, where one root came from the medial cord and three roots came from the lateral cord. Ghosh et al. [12] reported anomalies of three roots (one from medial and two from lateral), four roots (one from medial and three from lateral), and another four roots (one from medial, one from lateral, and two from posterior). Sharma et al. [13] reported the median nerve passing below the ulnar head of the pronator teres in 7.40% of their upper limb subjects, while a normal path between the two heads were seen in the other 92.60%. Caetano et al. [14] reported similar median nerve variation below pronator teres muscle in 3.48% of their subjects. The variations that were reported in the origin of the median nerve and the course of this nerve within the pronator teres muscles do not relate to the findings in our case in any way, but it serves as a record that variations of these structures did exist as described above.

Accessory brachial arteries emerging from the axillary artery and their reunion with the main brachial artery in the cubital fossa were reported as 7.14% by Chakravarthi et al. [15] and 1.25% by Kachlik et al. [16]. In our case, the “superficial” brachial artery found did not reunite with the “main” brachial artery but continued down the forearm and hand as the radial and ulnar arteries. Chakravarthi et al. also reported an unusual bilateral accessory brachial artery arising from the axillary artery in 4.29% of the cadavers [15]. Although very similar to the variation of the brachial artery in our case, Chakravarthi et al. [15] noted that the superficial artery as an early division of the ulnar artery as opposed to the radial artery. Chakravarthi et al. [17] reported the continuation of the superficial brachial artery as the radial artery, and D’Costa et al. [18] reported a unilateral superficial brachial artery that divided into superficial radial and superficial ulnar arteries.

Supracondylar fractures and their treatment within the pediatric age group have been well described by Lins et al. [19]. Supracondylar fractures involve three types, of which type III is of significance as it involves the displacement of the distal segment. All fractures with vascular impairment were treated with a closed reduction and percutaneous pinning [19]. In a person with a normal anatomy, the treatment outline might be applicable based on the vascular impairment, which includes diminished pulses, decreased capillary refill, and reduced pulse oximetry readings in regard to the radial artery distribution. However, such an injury to a person with variations such as our case could present with a normal radial pulse and might present with symptoms of severe median nerve and ulnar artery injuries. These two structures are normally protected from injury such as fractures, as they run on the surface of the brachialis muscle to enter the cubital fossa. In this case, the median nerve and ulnar artery are observed to run against the shaft of the distal humerus under the humeral head of the pronator teres. Supracondylar fractures can severely affect individuals with the kind of variations seen in our case.

Pronator teres syndrome, which was first described by Henrik Seyffarth in 1951, is primarily caused by the compression of the median nerve by the pronator teres muscle in the forearm [20]. The primary function of the pronator teres is pronation and actions, such as hammering, tennis, and so many other actions, which shows that this muscle extensively can cause hypertrophy, leading to the compression of the median nerve as it passes through the humeral and ulnar heads of this muscle. Sometimes, a fibrous band is said to make this compression worse [20]. With variations, such as those noted in our case, the probability of median nerve compression to be accompanied by ulnar artery insufficiency (due to constriction) can be high. This may lead to ischemia or compartment syndrome. Compression of the median nerve at a proximal level within the hypertrophied pronator teres can mimic symptoms of carpal tunnel syndrome. Thorough investigations are necessary to rule out all possibilities of variations before initiation of treatment. 

The elbow AVFs are a reliable means of establishing vascular access for hemodialysis. In a construction of 272 AVFs, Elcheroth et al. [21] talked about creating fistulas mainly between the brachial artery and a forearm vein for chronic hemodialysis purpose. Some AVFs were created between the brachial artery and cephalic vein or basilic vein. An anatomical variation may lead to confusion due to the absence of normal vessels in the cubital fossa, while raising the possibility of complications due to a more proximal location of the radial and ulnar arteries.

Cubital tunnel syndrome is the second most common entrapment neuropathy [22]. The ulnar nerve entrapment is relieved by in situ decompression and medial epicondylectomy. About 8 mm of the medial epicondyle of the humerus is resected and an anterior transposition of the ulnar nerve is performed to release the nerve from entrapment [22]. This kind of procedures can affect individuals with variations in the origin of pronator teres and with median nerve and ulnar artery coursing close to the shaft of lower humerus. Apart from the conditions discussed above, there could be other pathological inflammatory and ischemic conditions that involve the elbow joints and injuries involving head of radius and nearby structures. Therefore, whether it is trauma, disease conditions, or elective surgical procedures, variations around this region should always be in the forefront of a physician’s mind.

## Conclusions

Although several studies have disseminated information regarding high dividing brachial artery or variations in the formation of the median nerve, bilateral upper arm median nerve and brachial artery variations associated with a muscular anomaly as described in this case report have not been reported so far in the literature. As these variations carry both biomechanical and clinical implications, we urge clinicians to be aware of variations such as these when treating conditions that could arise due to compromise to neurovascular structures located within this region.
